# Mechanisms of stem cell based cardiac repair-gap junctional signaling promotes the cardiac lineage specification of mesenchymal stem cells

**DOI:** 10.1038/s41598-017-10122-6

**Published:** 2017-08-29

**Authors:** Heiko Lemcke, Ralf Gaebel, Anna Skorska, Natalia Voronina, Cornelia Aquilina Lux, Janine Petters, Sarah Sasse, Nicole Zarniko, Gustav Steinhoff, Robert David

**Affiliations:** 1 Reference- and Translation Center for Cardiac Stem Cell Therapy (RTC), Department of Cardiac Surgery, University of Rostock, Schillingallee 69, 18057 Rostock, Germany; 2 Faculty of Interdisciplinary Research, Department Life, Light & Matter, University Rostock, Albert-Einstein Str. 25, 18059 Rostock, Germany

## Abstract

Different subtypes of bone marrow-derived stem cells are characterized by varying functionality and activity after transplantation into the infarcted heart. Improvement of stem cell therapeutics requires deep knowledge about the mechanisms that mediate the benefits of stem cell treatment. Here, we demonstrated that co-transplantation of mesenchymal stem cells (MSCs) and hematopoietic stem cells (HSCs) led to enhanced synergistic effects on cardiac remodeling. While HSCs were associated with blood vessel formation, MSCs were found to possess transdifferentiation capacity. This cardiomyogenic plasticity of MSCs was strongly promoted by a gap junction-dependent crosstalk between myocytes and stem cells. The inhibition of cell-cell coupling significantly reduced the expression of the cardiac specific transcription factors NKX2.5 and GATA4. Interestingly, we observed that small non-coding RNAs are exchanged between MSCs and cardiomyocytes in a GJ-dependent manner that might contribute to the transdifferentiation process of MSCs within a cardiac environment. Our results suggest that the predominant mechanism of HSCs contribution to cardiac regeneration is based on their ability to regulate angiogenesis. In contrast, transplanted MSCs have the capability for intercellular communication with surrounding cardiomyocytes, which triggers the intrinsic program of cardiogenic lineage specification of MSCs by providing cardiomyocyte-derived cues.

## Introduction

Myocardial transplantation of adult stem cells offers a promising opportunity for cardiac regeneration and re-growth of irreversibly damaged tissue following myocardial infarction (MI) However, the beneficial effect is mostly limited (~3–5% functional improvement) and obtained results are often inconsistent^1–3^.

Selection of the optimal cell population for transplantation is one of the strategies currently explored to overcome the problems of cell therapeutics^4^. Among others, two major subtypes of cells isolated from BM are applied – hematopoietic stem cells (HSCs) and mesenchymal stem cells (MSCs)^4^. In the present study, we evaluated the potential benefit of co-transplantation of these two distinct cell populations. In particular, human CD271^+^ MSCs and CD133^+^ HSCs were injected into myocardium of immunodeficient mice after MI. Moreover, the difference between the underlying regenerative mechanisms of these cell types was investigated.

Another possible improvement strategy for stem cell therapeutics implies the enhancement of cell properties. This requires a comprehensive understanding of the mechanisms that govern the regenerative capacity of transplanted stem cells: direct (i.e. by engraftment, differentiation into myocardial or vascular lineages) and indirect (e.g. by activating other cells, cell-cell interaction, paracrine signaling, immunomodulatory effects, cell fusion, and the regulation of resident cardiac stem cell niches)^5, 6^. Manipulation of one of these – transdifferentiation – has already been proven successful in the recent phase II clinical trial C-CURE (NCT00810238). It showed feasibility and safety of lineage-guided stem cells (human MSCs exposed *ex vivo* to growth factors mimicking natural cardiogenic cell conversion) and a positive impact on cardiac performance vs. untreated cells^7^. The rapid clinical translation of this concept was mainly ensured by the *in vivo* success of these “next generation” stem cell products, based on genetic modification and cell preconditioning, including their transformation to cardiac progenitors prior to transplantation. For example, human BM derived stem cells were shown to undergo cardiac specification after stimulation with several trophic factors like TGF-β or BMP, triggering the expression of NKX2.5, GATA-4, Mef2C and other cardiac-specific proteins^7–9^. Subsequent animal studies in a murine model confirmed their enhanced regenerative potential^10^. Notably, apart from artificially guided cellular plasticity, cardiac lineage specification of stem cells has also been described to be an intrinsic event that is induced when cells are integrated into a cardiac environment^11–14^. Precise knowledge about these endogenous mechanisms will help to identify novel strategies for manipulation of cells in order to enhance their cardiac differentiation potential for clinical application e.g., by activation of their intrinsic transdifferentiation program.

Gap junctional intercellular communication (GJIC) between stem cells and cardiac cells was found to support the differentiation into cardiac progenitors^15–17^. Gap junctions (GJ) are specialized cell-cell contacts that allow the direct transfer of molecules between adjacent cells up to a molecular weight of 1.5 kD, including ions, metabolites and small non-coding RNA^18–20^. It has been recently described that endogenous regulation of stem cell fate is ensured by the surrounding cardiac tissue^21^. Similar mechanisms might be involved in the regulation of the fate of transplanted cells by the host myocardium.

In order to address this issue, we established an *in vitro* co-culture system composed of stem cells and cardiomyocytes (CM) to elucidate the role of gap junctional coupling in lineage specification of stem cells within a cardiac environment. While HSCs failed to establish functional GJs with adjacent myocytes, MSCs were found to successfully integrate into the CM monolayer in a GJ-dependent manner. The coupling activity was associated with an increased expression of NKX2.5 and GATA-4, indicating the cardiogenic differentiation of MSCs. These cardiac specific transcription factors were also found in MSCs after transplantation into mice hearts. Interestingly, this lineage specification might be supported by a gap junctional transfer of CM-derived miRNAs into MSCs.

In summary, our data suggest that the capability of certain stem cells to establish GJIC with myocytes favors their differentiation into cardiac progenitors and defines thereby the prevailing mechanism of their activity. The GJ-dependent shuttling of cardiogenic cues promoted the activation of the intrinsic trans-differentiation pathway of MSCs. Hence, GJs represent a promising target for the improvement of stem cell based therapies.

## Results

### Isolation and characterization of CD271^+^ MSCs and CD133^+^ HSCs

**Figure 1:**
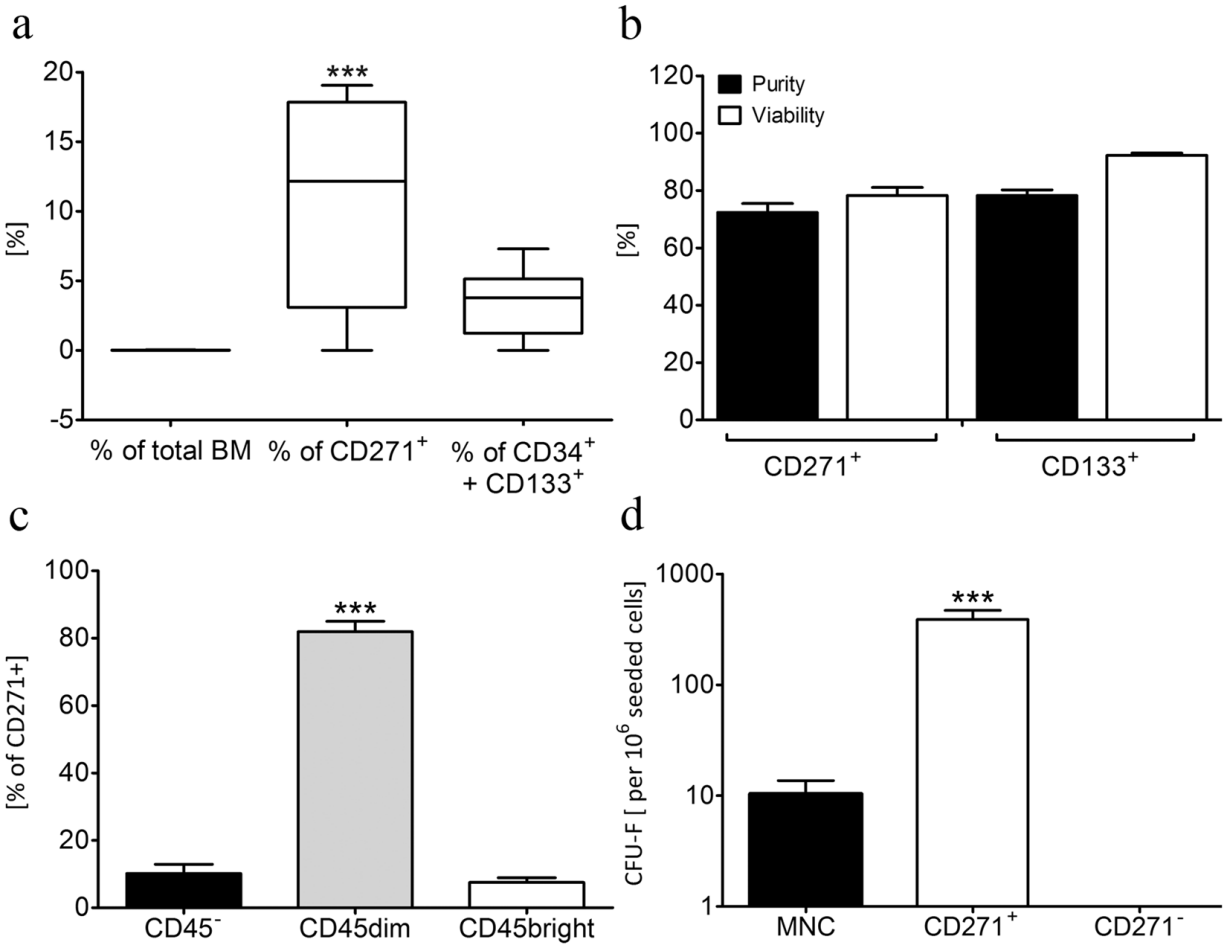
Flow cytometric characterization of CD271^+^ and CD133^+^ stem cell populations in human BM. (**a)** Population overlap of CD271^+^/CD133^+^ cells was determined in total BM, CD271^+^ and CD133^+^ subpopulations, respectively. Boxes enclose 25% - 75% values with median line, each outlier is displayed, n = 6. (**b)** Cell purity and viability of MACS-isolated CD271^+^ (n = 44) and CD133 + cell fractions, n = 75. (**c)** Analysis of CD45 expression of purified CD271^+^ cells revealed distinct subpopulations. The high expression of CD45^dim^ demonstrates that mesenchymal stem cells represent the major cell population within the CD271^+^ fraction, n = 40. (**d)** CFU-F assay of mononuclear cells, purified CD271^+^ cells and CD271^−^ fraction confirmed that all mesenchymal progenitors originated from CD271^+^ cells, n = 11. Graphs are shown as mean ± SEM. Statistical differences were analyzed using one-way ANOVA, followed by Tukey’s post hoc test (***P < 0.0001).

We intended to study the therapeutic capacity of CD271^+^ and CD133^+^ BM derived stem cells independently and in synergy, upon simultaneous injection. In such an experimental setup, the efficient isolation of these cell types is a prerequisite. Therefore, we established a flow cytometric strategy to evaluate a possible population overlap of CD133^+^ and CD271^+^ cells that might impede sufficient purification of respective cell fractions (Supplementary Fig. S1). Initially, the frequency of CD271^+^/CD133^+^ cell subpopulation was analyzed in total BM and we found that 0.017 ± 0.006% expressed both CD133 and CD271 surface markers (Fig. 1a). Further, we evaluated the percentage of this double positive cell subset either within the CD271^+^ or CD133^+^ cell subpopulation. This precise marker analysis revealed that a CD271^+^/CD133^+^ cell subpopulation was significantly more frequent in CD271^+^ cells than in CD133^+^ cells (Fig. 1a). Due to the fact that only a small percentage of isolated cells express both markers, we estimated the separation of the two populations to be sufficient for the evaluation of their separate or combined effects.

Next, we investigated the quality of MACS-based cell isolation by evaluating purity and viability of each cell fraction. Isolated CD133^+^ cells were analyzed using an adapted ISHAGE gating strategy for CD34^+^/CD133^+^ cells^22^. For analysis of the CD271^+^ fraction, cells were stained for CD271 and CD45 surface markers. Flow cytometric measurements revealed a mean purity and viability of 78.2 ± 2.0%/92.2 ± 0.8% for CD133^+^ cells and 72.4 ± 3.1%/78.3 ± 2.8% for CD271^+^ cells (Fig. 1b).

In contrast to CD133^+^ stem cells, CD271^+^ cells have been described to contain all mesenchymal colony forming progenitors^23^. Since a reduced expression of CD45 is a common hallmark of mesenchymal progenitor cells^24^, MACS-purified CD271^+^ cells were characterized for different CD45 expression levels (negative (−), dim, bright). We found that CD45^dim^ represented the major cell population (Fig. 1c). Further, we conducted a CFU-F assay, showing the formation of colonies in mononuclear cells that resided exclusively in the CD271^+^ fraction. Importantly, CD271^+^ purification dramatically increased the amount of colonies, while no colonies were observed in the CD271^−^ fraction. Together, these results give evidence that the most frequent population in purified CD271^+^ cells in our study mainly consists of MSCs (Fig. 1d).

Compared to freshly isolated CD271^+^ cells, expanded MSCs demonstrated a similar expression level of CD271 (freshly isolated CD271^+^ MSCs vs. expanded MSCs: 72.37 ± 3.09% vs. 64.7 ± 12.2%), while an increased expression of relevant mesenchymal markers (CD105, CD44, CD73, CD45) was detected (freshly isolated CD271^+^ MSCs vs. expanded MSCs: 1.2 ± 0.3% vs. 78.9%).

Additionally, to investigate whether MSCs might possess pericytic properties, freshly MACS-isolated CD271^+^ cells were analyzed using pericyte specific markers, including CD146, PDGFR-β, and CD34, CD45^25^. Flow cytometry analysis of viable mononuclear cells of the CD271^+^ fraction revealed that 0.062 ± 0.014% are CD271^+^/CD146^+^/CD45^−^ and only 0.033 ± 0.005% of these cells are corresponding to the CD271^+^/CD146^+^/CD45^−^/PDGFR-β^+^/CD34^−^ pericytic phenotype. Moreover, the frequency of pericytes within the CD271^+^ cell fraction increased upon cultivation of MSCs (43.4%).

### Single cell treatment and mixed application of MSCs and HSCs improve cardiac functions

**Figure 2:**
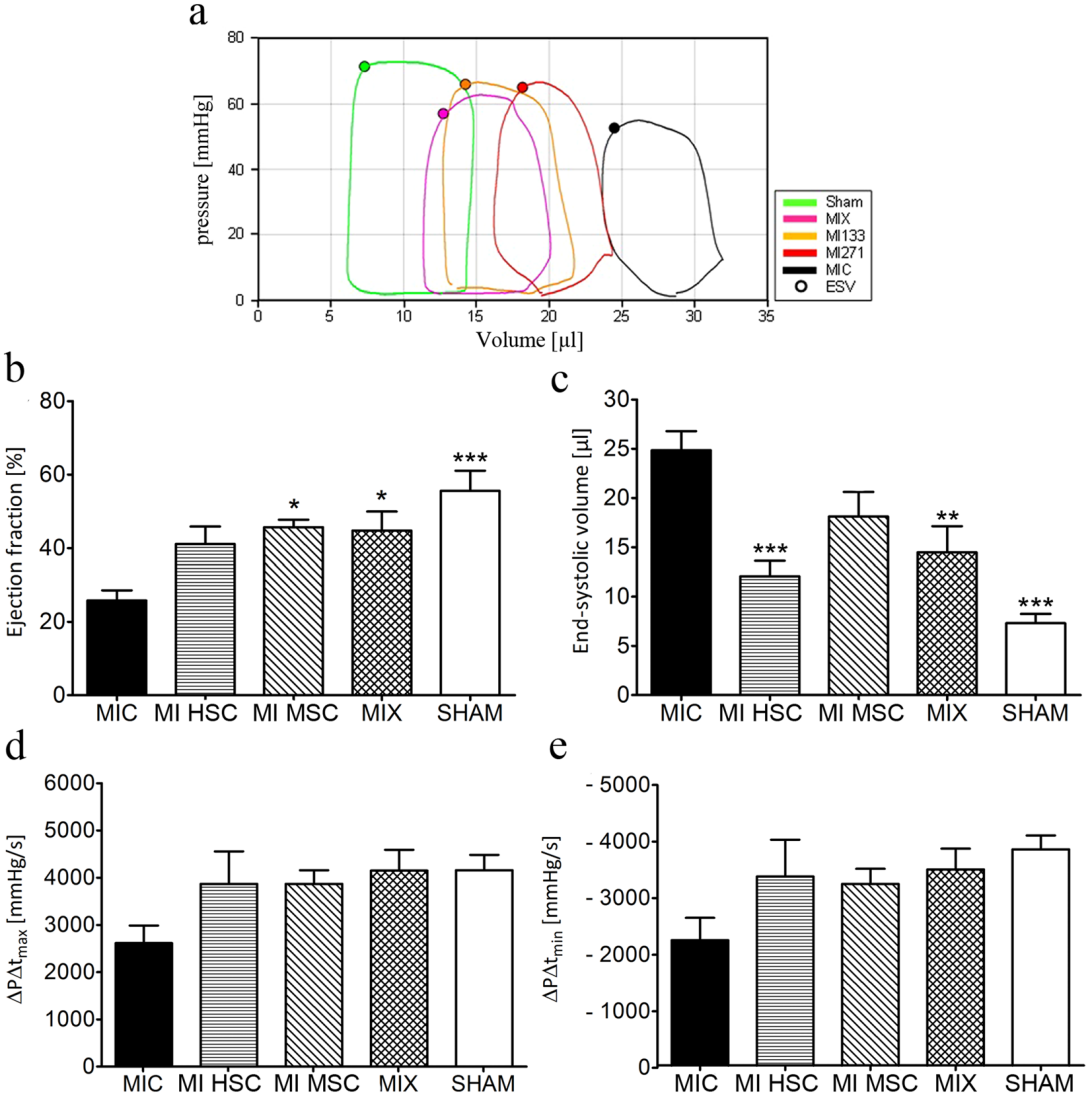
Therapeutic effect of MSCs and HSCs treatment on cardiac functions. (**a**) Representative pressure volume (PV)-loop graph used to evaluate cardiac performance. (**b**,**c)** Compared to untreated MI (MIC), ejection fraction and end-systolic volume were significantly improved when MSC and HSC populations were applied, both as single injection (MI HSC, MI MSC) and co-administration (MIX), n = 7. (**d**,**e)** Likewise, PV-loop analysis showed that stem cell treated hearts demonstrated an improved velocity of pressure rise (max/min), compared to the untreated MIC group, n = 7. Graphs are shown as mean ± SEM. Statistical differences were analyzed using one-way ANOVA, followed by Tukey’s post hoc test (*P < 0.05, **P < 0.01, P < 0.001), vs. MIC.

Next, we assessed the impact of stem cell transplantation on cardiac performance after myocardial infarct. To evaluate this cardiac regenerative efficiency of isolated CD271^+^ MSCs and CD133^+^ HSCs, 10^5^ cells were transplanted into SCID *beige* mice after cardiac ischemia/reperfusion by short-time ligation of the left anterior descending artery (LAD), followed by pressure volume loop analysis (Fig. 2a). Compared to untreated MI (MIC), human stem cell transplantation induced a significant improvement of the ejection fraction (EF) 3 weeks after cell transplantation. We could not observe differences in the functional improvement between these two stem cell treated groups (Fig. 2b). Analysis of the animals with co-transplanted MSCs and HSCs (MIX) revealed comparable positive effects on EF. Additionally, analysis of the end-systolic volume and contractility of the heart confirmed the benefits of MSC and HSC treatment on cardiac performance (Fig. 2c–e). The pressure-volume loop data showed that co-transplantation induced a slight but non-significant improvement compared to single treatments (Fig. 2d,e).

### Simultaneous stem cell transplantation is beneficial in terms of MI-induced cardiac remodeling and neovascularization compared to single treatment

**Figure 3:**
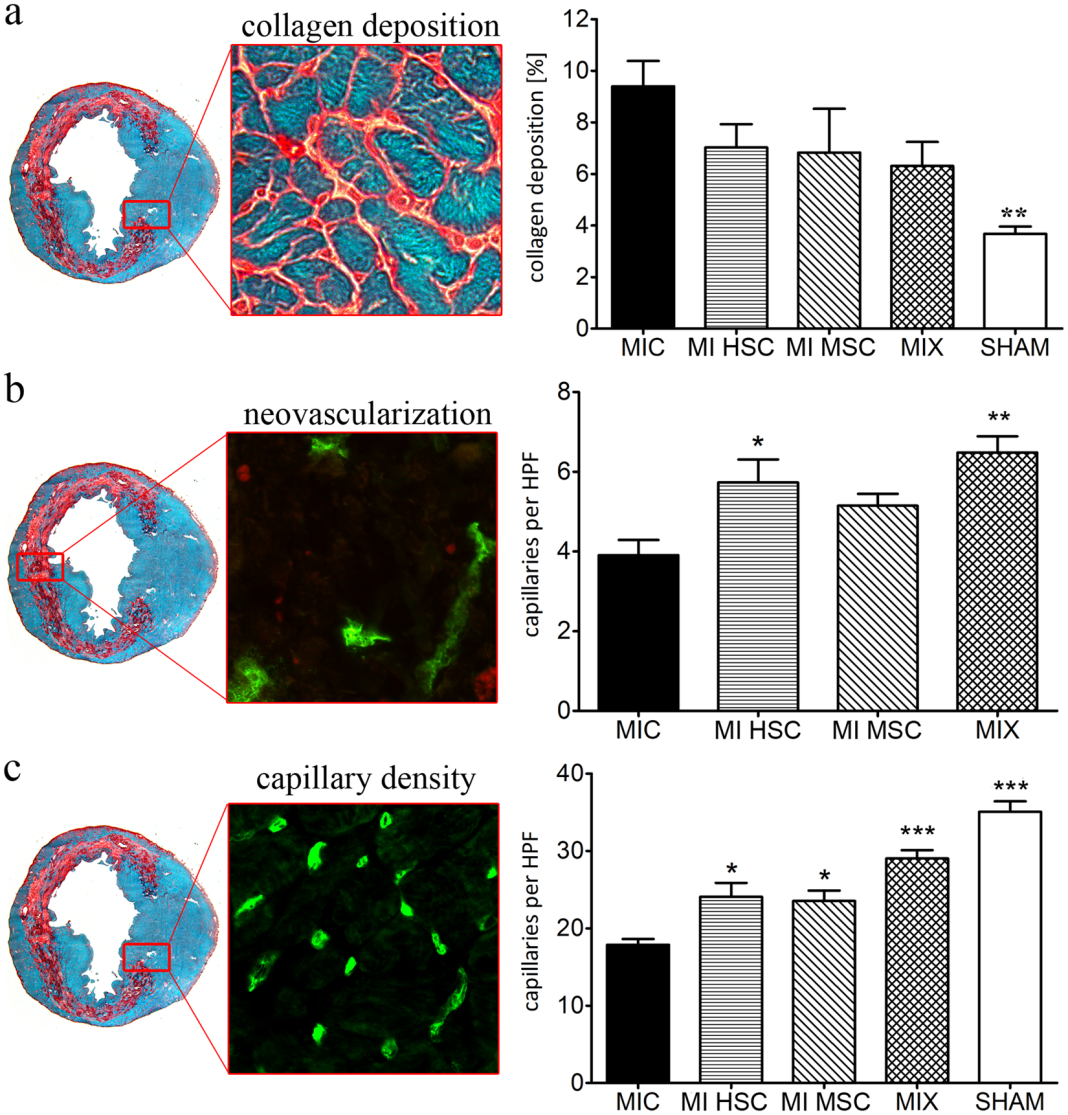
Impact of MSCs and HSCs on MI-induced cardiac remodeling and neovascularization defined for co-transplanted cells and single populations. **(a)** Stem cell treatment (MSC, HSC, MIX) decreased collagen deposition at the infarction border in comparison to untreated MI (MIC), n = 7. (**b)** Effect of stem cell transplantation on neovascularization determined within the infarcted area. Infiltration of the infarcted scar with new blood vessels was improved in stem cell treated hearts compared to MIC, n = 7. (**c)** The capillary density in the border zone was significantly higher following single injection or co-administration of MSCs and HSCs, in contrast to untreated MIC, n = 7. Graphs are shown as mean ± SEM. Statistical differences were analyzed using one-way ANOVA, followed by Tukey’s post hoc test (*P < 0.05, **P < 0.01, P < 0.001), vs. MIC.

To address the impact of stem cell transplantation on MI-induced cardiac remodeling we investigated two of its indicators, capillary density and collagen deposition at the border zone of the infarcted region. While untreated MI demonstrated the strongest accumulation of collagen, application of human stem cells decreased collagen deposition (Fig. 3a).

The influence of stem cell therapy on the extent of neovascularisation was addressed by determination of the capillary density within the infarcted area. Since the healthy heart tissue contains both old and newly formed vessels, neovascularization can only be properly evaluated within the fibrotic region. A distinctly higher number of vessels were found in animals that have undergone stem cell treatment, both, single and co-injected (Fig. 3b). This increased density of capillaries is also reflected in the functional tissue at the border zone (Fig. 3c). Both MSCs and HSC treated animals demonstrated a significantly higher amount of vessels compared to MIC (Fig. 3c). Interestingly, this positive effect was even more pronounced in the MIX group.

**Figure 4:**
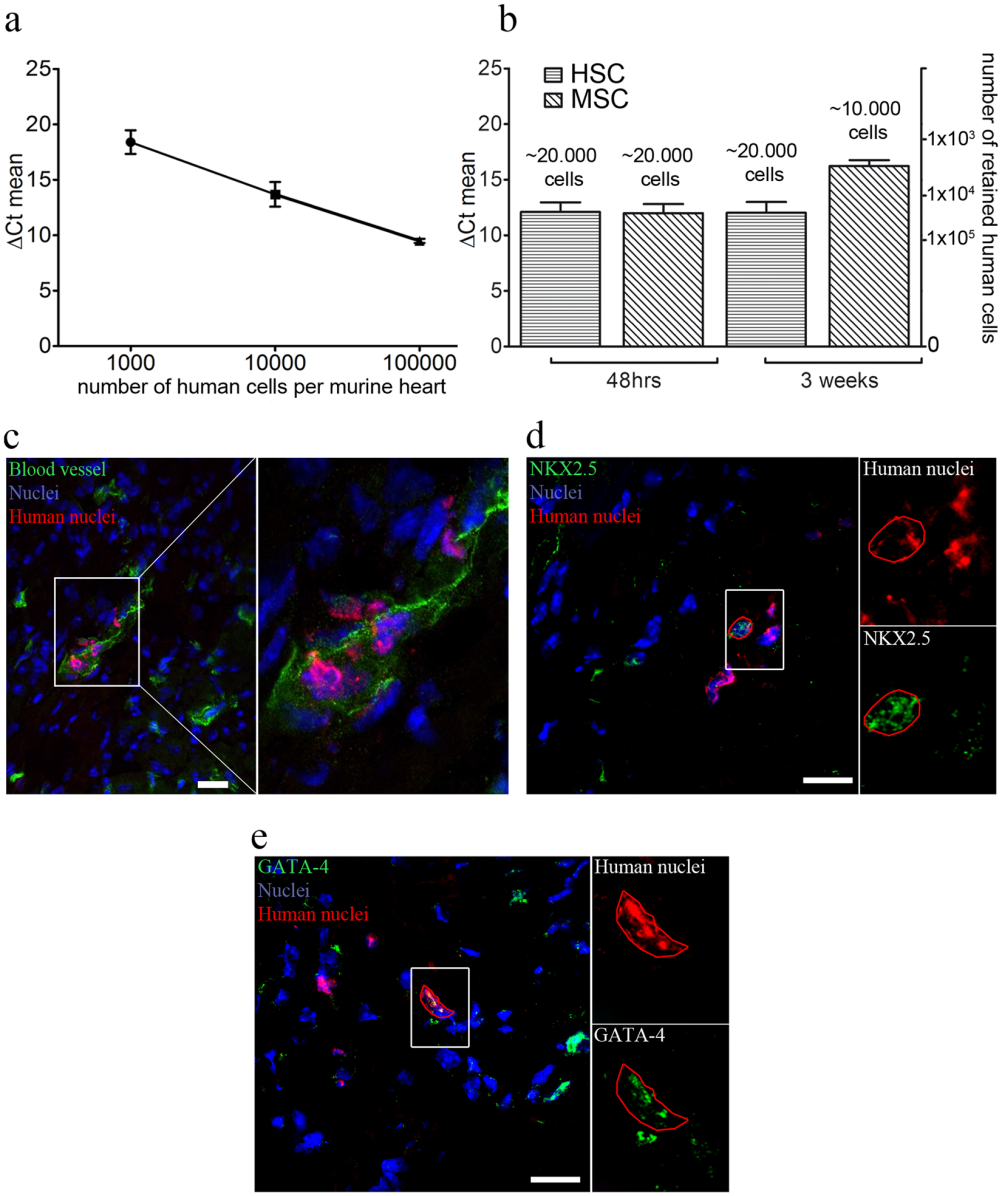
Retention, engraftment and therapeutic activity of MSCs and HSCs *in vivo*. (**a)** Sensitivity test of qRT-PCR with stem cell treated cryosectioned hearts revealed a detection threshold of 1 × 10^3^ cells using human GAPDH, n = 6. (**b)** While the number of retained cells per murine heart was similar 48 h after injection, HSCs demonstrated an increased retention rate three weeks post infarction compared to MSCs, n = 6. (**c)** Immunostaining of HSCs in stem cell-treated cryosectioned hearts gave evidence for the co-localization of HSCs with tomato-lectin labeled blood vessels. No co-localization with vessels was found for MSCs. (**d**,**e)** Following transplantation into mice hearts, MSCs demonstrate cardiac specific lineage specification as shown by labeling of the cardiac specific transcription factor NKX2.5 and GATA-4. Scale bars 20 µm. Graphs are shown as mean ± SEM.

To verify whether the improvement of cardiac function and MI-induced cardiac remodeling is linked to the transplanted MSCs and HSCs resided in the infarcted heart, qRT-PCR of cryosectioned heart and lung tissue was performed to identify the fate of injected stem cells. qRT-PCR allowed us to achieve a detection limit of at least 1000 cells by determination of a detection threshold using human GAPDH (Fig. 4a, Supplementary Table S1). The amount of both, MSCs and HSCs, in the lung was below the detection limit after 48 h in 50% of all treated animals and after 3 weeks in 100% of all treated animals (data not shown). In contrast, we detected stem cell derivatives in the infarcted heart up to 3 weeks after transplantation. While no difference in cell retention was observed 48 h after transplantation, the retention of HSCs was slightly higher compared to MSCs 3 weeks after infarction (Fig. 4b, Supplementary Table S1). The entire list of all delta Ct values of performed qRT-PCRs can be found in Supplementary Table S1.

Histological analysis of cryosectioned hearts further revealed a difference in the therapeutic activity of MSCs and HSCs. Additional staining of perfused blood vessels with tomato lectin showed the co-localization of HSCs with functional blood vessels (Fig. 4c). This direct impact of HSCs on angiogenesis is well documented and was not detected for MSCs, suggesting different cellular mechanisms for cardiac regeneration. For MSCs, transdifferentiation was shown to be a possible key mechanism that might explain their positive therapeutic effects. Indeed, transplanted MSCs were found to express NKX2.5 and GATA-4, two transcription factors that are attributed to the early stage of cardiac development, indicating a transdifferentiation capacity of MSCs *in vivo* (Fig. 4d,e, Supplementary Fig. S2).

Taken together, our *in vivo* data indicate that the application of MSCs and HSCs counteracts pathological alterations that occur during the remodeling process following MI, including vascularization and collagen deposition. Moreover, these beneficial effects correspond to their retention in the infarcted tissue. Interestingly, the specific regenerative mechanism of HSCs seems to differ from that of MSCs. The possible underlying reasons for this difference were addressed in extensive subsequent *in vitro* experiments simulating cardiac environment.

### MSC and CM communicate via functional gap junctions

**Figure 5:**
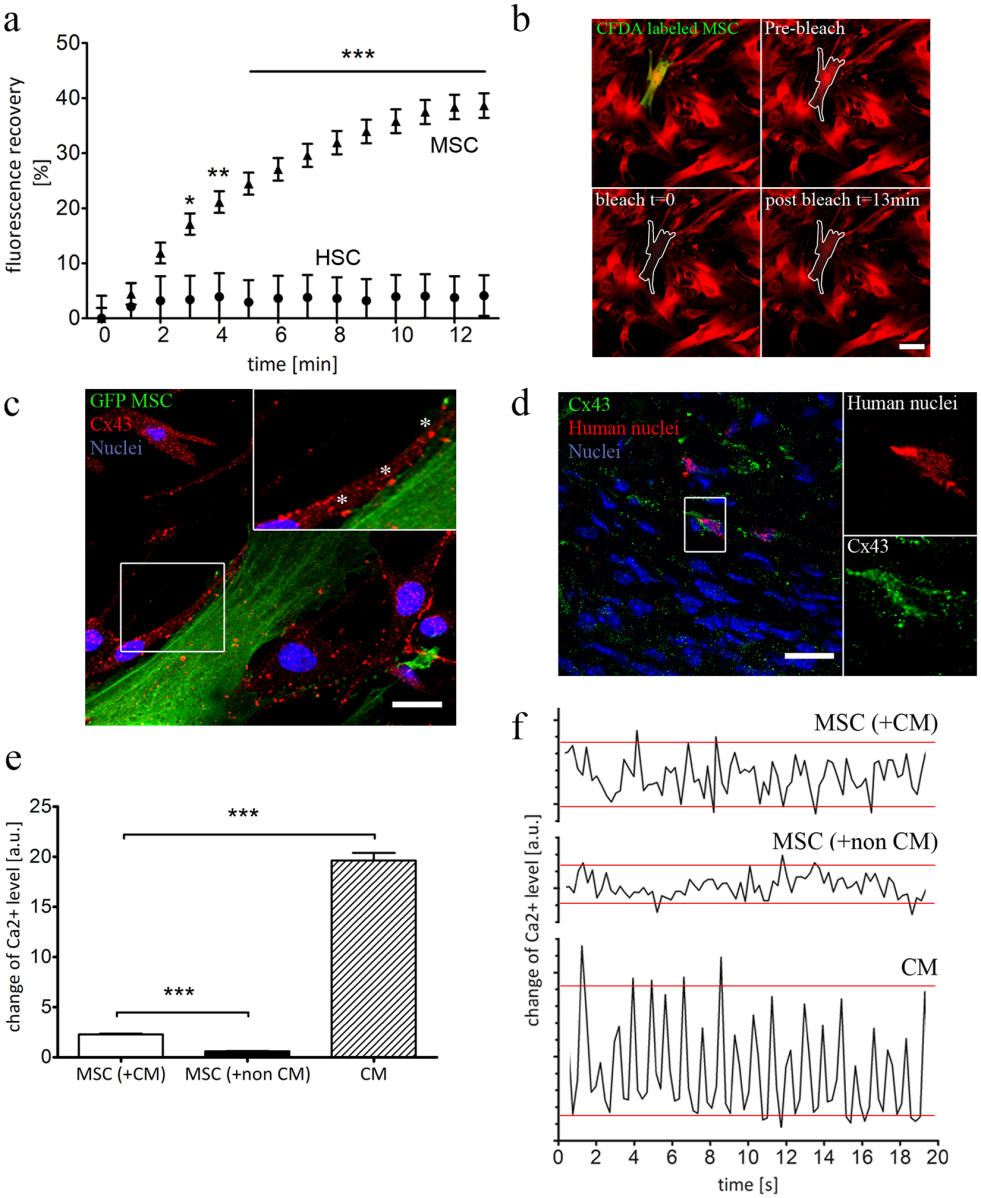
Establishment of functional GJs between MSCs and CMs. (**a)** MSCs and HSCs were co-cultured with cardiomyocytes, loaded with calcein and subjected to FRAP microscopy. While MSCs demonstrated strong fluorescence recovery, no recovery was observed for HSCs, n = 3, 38 cells. (**b)** Representative FRAP images demonstrated the influx of calcein from surrounding CMs into the bleached MSC (CFDA labeled), indicating the presence of functional GJs (**c)** Immunolabeling of MSC-CM co-cultures revealed the presence of Cx43 plaques (*) at cell borders between GFP-labeled MSC and CMs. (**d)** A strong cytoplasmic expression of Cx43 in MSCs was also confirmed *in vivo*, following injection into mice heart. (**e)** Gap junctional coupling with CMs induced Ca2^+^ transients in MSCs. Changes of the intracellular Ca2^+^ level were visualized using X-Rhod-1. Compared to co-culture with non-CMs, co-cultivation with CMs induced significant stronger changes of the intracellular Ca^2+^ level in MSCs. As expected, the alterations of intracellular Ca^2+^ were more pronounced in CMs, n = 3. (**f)** Representative plots of Ca^2+^ level changes. The amplitude of the oscillating signal of X-Rhod-1 in MSCs is higher when cells are co-cultured with CMs, compared co-culture with non-CMs. However, these Ca^2+^ transients are less distinct and demonstrated a more irregular pattern than Ca^2+^ transients found in CMs. Scale Bars 20 µm, Graphs are shown as mean ± SEM. FRAP curves were statistically analyzed by two-way ANOVA (*P < 0.05, **P < 0.01, ***P ≤ 0.001), followed by Bonferroni’s post-hoc test. Statistical significance between Ca^2+^ levels was analyzed using Statistical differences were analyzed using one-way ANOVA, followed by Dunn’s post hoc test (***P < 0.001).

GJs are specialized cell-cell contacts that allow the direct exchange of signaling molecules between the host myocardium and transplanted cells, which might alter the phenotype and physiology of transplanted stem cells, e.g. their differentiation status. Therefore, we studied the possible role of gap junctional coupling in the above observed therapeutic effects of transplanted stem cells and the difference in their regenerative activity. For this purpose, we used an *in vitro* co-culture system composed of MSCs or HSCs and neonatal CMs. First, we investigated the ability of MSCs and HSCs to establish functional GJs with adjacent myocytes. To determine GJ activity, co-cultures of CMs and CFDA-labeled MSCs or HSCs were loaded with a red fluorescent GJ-permeable calcein dye and subjected to FRAP microscopy. Photobleaching of MSCs resulted in a fluorescence recovery of 39 ± 2.2%, indicating a high gap junctional influx of dye molecules from adjacent CMs (Fig. 5a,b). On the contrary, HSCs did not show fluorescence recovery when subjected to photobleaching, indicating the lack of gap junctional cell-cell contacts to adjacent myocytes (Fig. 5a). These observations were confirmed by analysis of the expression of Cx43 protein, the main building block of GJs in the mammalian adult working myocardium. For MSCs, confocal imaging revealed that both CMs and MSCs express Cx43, which was visualized as large dots between these two cell types, suggesting the presence of GJs (Fig. 5c). Moreover, a significant cytoplasmic expression of Cx43 was also demonstrated *in vivo* as shown by immunofluorescence labeling of cryosectioned hearts (Fig. 5d), while no fluorescence signal was detected for HSCs (data not shown).

As known from previously published studies, cardiac transdifferentiation is one mechanism of MSCs’ activity that seems to be triggered by an intercellular crosstalk with the host tissue. Since the successful gap junctional coupling of stem cells with CMs was shown only for MSCs (but not HSCs), we focused on this particular stem cell type to study the impact of GJIC on cardiac lineage specification.

In order to further prove the establishment of functioning GJIC between MSCs and CMs, the regulation of the intracellular Ca^2+^ level was examined. ±We used X-Rhod-1, a Ca^2+^-sensitive dye that demonstrates an increase of fluorescence intensity upon Ca^2+^ binding, leading to an oscillating fluorescence signal when calcium transients are present. Interestingly, we found that MSCs co-cultured with CMs showed a 3.8-fold higher amplitude of the oscillating X-Rhod-1 signal than MSCs cultured with non-cardiomyocytes (Fig. 5e, Supplementary movie S1). Yet, compared to CMs, the amplitudes of X-Rhod-1 intensity in MSCs were considerably lower (Fig. 5e). This is clearly illustrated by representative plots of the oscillating signal of X-Rhod-1 (Fig. 5f, red lines). Moreover, co-cultured MSCs demonstrated a less pronounced and irregular frequency pattern compared to CMs.

These results suggest that transplanted MSCs have the potential to be integrated into the host cardiac tissue and to exchange signaling molecules with adjacent myocytes via GJIC. However, the role of such cell-cell connections in the transdifferentiation (i.e. NKX2.5 and GATA-4 expression) remains to be defined.

### GJs promote the expression of cardiac-specific markers in MSCs

**Figure 6:**
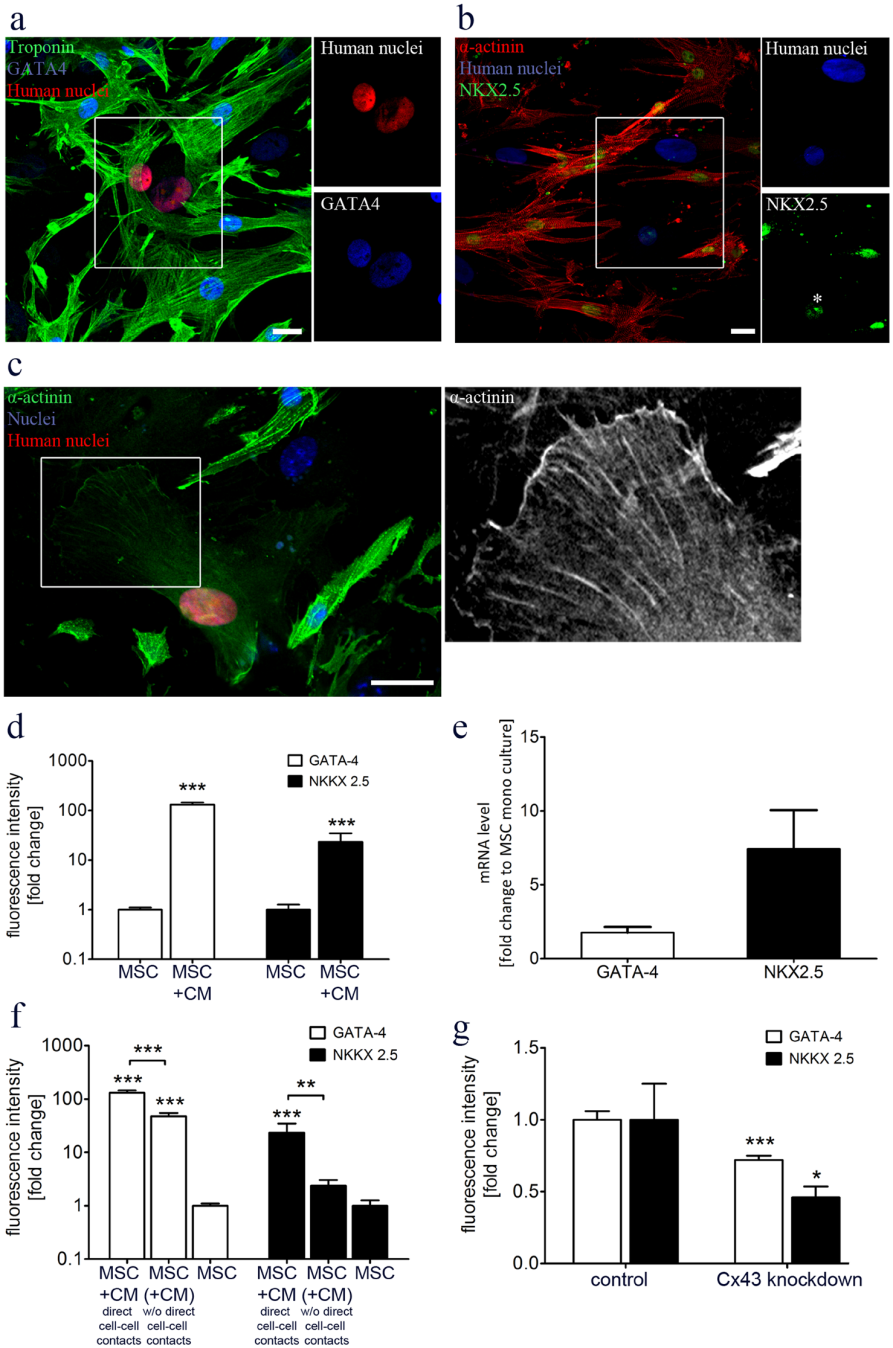
Gap junctional coupling with CMs promotes the cardiogenic differentiation of MSCs. (**a**,**b)** Immunofluorescence labeling of cardiac specific markers revealed the transdifferentiation of MSCs into a cardiac-like phenotype. MSCs co-cultured with CMs showed expression of GATA-4 and NKX2.5, two early cardiac specific transcription factors. (**c)** MSCs were found to express α-actinin, indicating the formation of pre-sarcomeric structures. (**d)** Quantitative assessment of NKX2.5 and GATA-4 expression by fluorescence microscopy demonstrated a marked increase when MSCs were co-cultivated with CMs, n = 3, 175 cells, note the logarithmic scale. (**e)** qRT-PCR confirmed the pronounced up-regulation of NKX2.5 and GATA-4 induced by co-culture with CMs, compared to MSC mono-culture, n = 3. (**f)** The establishment of cell-cell contacts between MSCs and CMs resulted in a significant higher protein level of GATA-4 and NKX2.5 compared to MSCs that share the same medium but lack direct cell-cell interaction with CMs, n = 3, 148 cells, note the logarithmic scale. (**g)** Downregulation of Cx43 in co-cultured MSCs reduced the expression levels of both transcription factors, indicating a promoting role of GJs in the transdifferentiation process of MSCs induced by adjacent CMs, n = 5, 409 cells. Scale Bars 20 µm, Graphs are shown as mean ± SEM. Statistical significance was analyzed using two-tailed Student’s t-test (*P ≤ 0.05, **P ≤ 0.01, ***P ≤ 0.001).

Next, we asked whether a cardiac environment might trigger the transdifferentiation of MSCs into a cardiac-like phenotype. Time-lapse live cell imaging showed that EGFP-labeled MSCs, integrated into a cardiac cell layer, were able to contract, consistent with the beating activity of surrounded CMs (Supplementary Fig. S3, Supplementary movie S2). However, it remained unclear whether the beating of MSCs was an active, α-actinin-mediated process or passive motion caused by adjacent, contracting CMs. Therefore, we investigated different cardiac markers to evaluate the differentiation status of co-cultured MSCs in more detail. Consistent with our *in vivo* data, MSCs were found to express NKX2.5 and GATA-4 upon co-culture with CMs (Fig. 6a,b). Moreover, MSCs expressed α-actinin, which suggests an early state of sarcomere formation that can provide the capacity of active cell contraction. However, z-disks were not observed, which likely reflects the immature state of co-cultured MSCs (Fig. 6c).

For quantitative assessment of the cardiogenic differentiation, we determined the expression levels of GATA-4 and NKX2.5 in human cell nuclei Compared to the control group, the expression levels of NKX2.5 and GATA-4 were markedly increased in MSCs that were co-cultured with CMs (Fig. 6d). The reliability of the microscopic analysis was confirmed by qRT-PCR, showing a pronounced increase of GATA-4 and NKX2.5 mRNA levels in MSCs upon co-culture with CMs (Fig. 6e).

To conclude, the data show that a cardiac environment triggers the transdifferentiation of MSCs. Although we already demonstrated a direct interaction between MSCs and CMs via GJs (Fig. 5a,c), paracrine events are known to influence stem cell differentiation as well^26^. To distinguish between these two possibilities, we compared the expression levels of NKX2.5 and GATA-4 between co-cultured MSCs and MSCs that share the medium with CM, but lack direct cell-cell interaction. The expression levels of GATA-4 and NKX2.5 were increased under both conditions when compared to independent MSC mono-culture. However, the establishment of cell-cell contacts between MSCs and CMs in co-culture led to a significant up-regulation of both transcription factors (Fig. 6f). In order to approve an involvement of gap junctional coupling in the cardiac lineage specification of MSCs, GJIC in co-cultured MSCs was impaired by siRNA mediated knockdown of Cx43. As a result, the expression levels of NKX2.5 and GATA-4 significantly decreased, which emphasizes the important role of functional GJs as mediators in the transdifferentiation process of MSC within cardiac tissue (Fig. 6g).

### GJ-dependent miRNA exchange between MSC and CM

**Figure 7:**
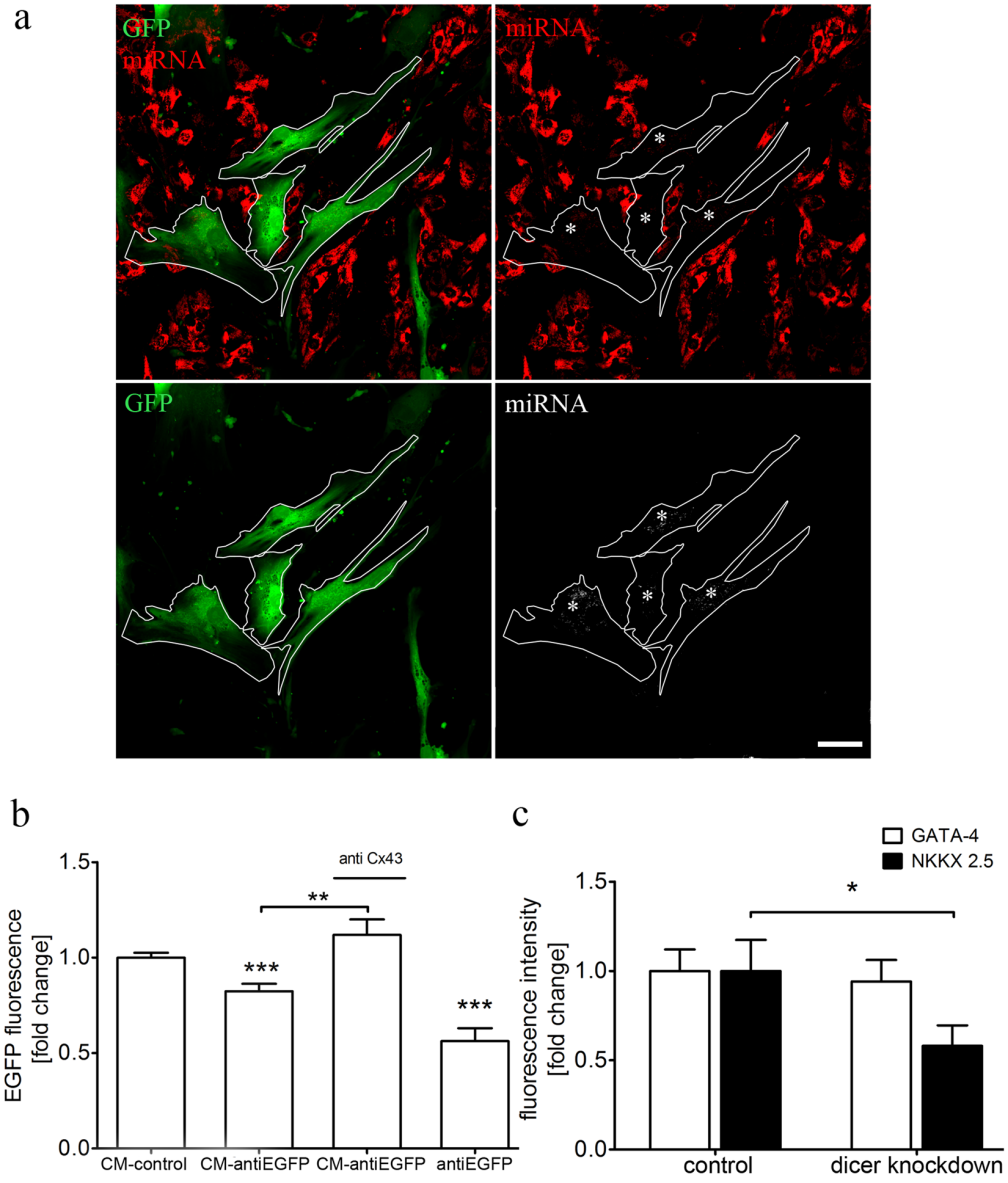
GJ-dependent delivery of small RNA from CM influences the transdifferentiation of MSCs. (**a)** EGFP-labeled MSCs were co-cultured with CMs transfected with DY-547 labeled miRNA. 24 h after co-cultivation, miRNA was also found in MSCs (asterisk), indicating the exchange of small RNAs between these two cell types, Scale bar 20 µm. (**b)** CM-derived small RNAs induce gene silencing in MSCs. MSCs were transfected with a plasmid coding for EGFP and co-cultured with CMs transfected with anti-EGFP siRNA. Following co-cultivation for 2 days, EGFP expression in MSCs was decreased by 20% when co-cultured with CMs containing anti-EGFP-siRNA. Downregulation of Cx43 in MSCs diminishes the EGFP-reducing effect of CMs. Efficiency of the EGFP/siRNA reporter construct was evaluated by double transfection of MSCs with EGFP and siRNA, resulting in a reduced EGFP intensity of ~50%. n = 3. (**c)** To verify the impact of CM-derived small RNAs on the transdifferentiation of MSCs, Dicer was down-regulated in CMs. Resulting disruption of the miRNA machinery in CMs impaired the transdifferentiation process in co-cultured MSCs. While NKX2.5 was significantly decreased in MSCs, no effect was observed on the expression level of GATA-4 upon co-cultivation with dicer knockdown CMs, n = 3, 255 cells. Graphs are shown as mean ± SEM. Statistical significance between EGFP fluorescence intensities was analyzed using one-way ANOVA, followed by Dunn’s post hoc test (**P < 0.01, ***P < 0.001). Statistical significance for GATA-4 and NKX2.5 levels was analyzed using two-tailed Student’s t-test (*P ≤ 0.05, ***P ≤ 0.001).

Since gap junctional coupling represents a pathway to transfer cardiac signaling molecules to transplanted MSCs we asked what kind of cardiac signals could be delivered by CMs to facilitate the cardiogenic differentiation of MSCs (Fig. 6). Small RNAs are very important regulators of development that were reported to be transferred via GJs leading thereby to altered gene expression in the recipient cell^27^. To test the capability of CMs to transfer small RNA into MSC, CMs were transfected with DY-547 tagged miRNA and co-cultured with EGFP-labeled MSCs for 24 h. Confocal microscopy revealed that labeled miRNA was also present in MSCs, indicating the shuttling of miRNA from CMs to MSC (Fig. 7a). This was further confirmed using functional siRNA as part of a EGFP/siRNA reporter system. While MSCs carried a plasmid coding for EGFP, CMs were transfected with a siRNA targeting the EGFP sequence. Following co-culture, the expression of EGFP in MSCs was significantly reduced by ~20%, suggesting a transfer of anti-EGFP siRNA from CMs into MSCs (Fig. 7b, Supplementary Fig. S4). In contrast, co-culture with control transfected CMs had no effect on EGFP intensity. The particular involvement of GJs in the transfer of small RNA from CMs to MSCs was investigated by Cx43 knockdown. The down-regulation of Cx43 in EGFP expressing MSCs abolished the ability of CMs to reduce EGFP expression. According to our results obtained by fluorescence microscopy (Fig. 7a), these data clearly indicate a transfer of small RNA, from CMs to MSCs in a GJ-dependent manner.

When CM-derived miRNAs facilitate the cardiac-lineage specification of MSCs, impairment of the miRNA machinery of CMs might affect this transdifferentiation process. Therefore, CMs were subjected to siRNA-mediated knockdown of Dicer, an RNAse that cleaves pre-miRNA into short ~22nt long duplexes. The resulting expression of NKX2.5 in MSCs strongly decreased by 40% when co-cultured with CMs with down-regulated Dicer RNAse (Fig. 7c). Interestingly, GATA-4 levels remained constant and were not influenced by Dicer knockdown. Thus, miRNA delivered from CMs, promotes the expression of NKX2.5. However, the level of GATA-4 was not affected which points to an involvement of other mechanisms regulating this transcription factor related to the cardiac differentiation of MSCs.

## Discussion

### Co-transplantation of HSCs and MSCs improves MI-induced cardiac remodeling

In the present study we investigated possible beneficial effects of co-transplantation of both human HSCs and MSCs on MI-induced mice. Clinical evidence indicated that intramyocardial transplantation of BM-derived stem cells in combination with coronary artery bypass grafting is associated with an improvement of functional parameters in patients with chronic ischemic heart disease^28–30^. Recent *in vitro* and *in vivo* data suggested a benefit of simultaneous transplantation of MSCs as an additive to allogenic HSC transplantation. In particular, MSCs were shown to promote HSCs recovery and decrease the incidence of acute graft-versus-host disease^31, 32^. However, the benefits of co-transplantation in MI-treated hearts are unknown and require further investigation. In our experimental model of MI, the injection of MSCs or HSCs alone significantly improved cardiac function. Notably, co-transplantation has resulted in an improved vascular density, neovascularization and reduced collagen deposition during MI-induced cardiac remodeling (Fig. 3), although no statistically significant synergistic effects on cardiac performance were observed (Fig. 2). These data are in agreement with clinical trials that also failed to demonstrate a clear benefit upon co-transplantation^33^.

### A cardiac environment triggers the transdifferentiation of MSCs

Animal studies demonstrated that MSCs are capable to acquire a CM-like phenotype^11, 26, 34^ leading to reduced infarction size and decreased fibrosis^35–37^. However, the ability of BM derived MSCs to transdifferentiate into functional CMs and to replace damaged heart tissue is controversially discussed^11, 14^. Increasing the frequency of this lineage conversion would lead to an improvement of stem cell treatments. Here, we showed *in vivo* that transplanted MSCs express the cardiac-specific transcription factors NKX2.5 and GATA-4 as well as α-actinin (Figs 4 and 6), which is in agreement with earlier reports^11, 15^. Hence, we conclude that the observed contractions of MSCs (Supplementary movie S2) could be partially based on active movement and in parts induced by the contraction of surrounding CMs. Similarly, a number of studies demonstrated that co-culture with CMs *in vitro* is not sufficient to induce a complete transdifferentiation of adult MSCs into functional CMs. However, it induces the activation of key cardiac specific genes, like NKX2.5, α-actinin and cardiac troponin, which provide evidence that the cardiac microenvironment strongly influences the plasticity and cell fate of transplanted MSCs^13, 14, 38, 39^.

Although this mechanism of cardiac lineage conversion contributes to the recovery of the heart after myocardial infarct, transdifferentiation of MSCs alone cannot explain the large beneficial effects of stem cell-based therapy. MSCs are also known to release various paracrine factors, such as VEGF or EGF, leading to neovascularization and endogenous cardiac regeneration^40–42^. Moreover, the anti-inflammatory property of MSCs is another important aspect for their successful use in regenerative medicine^43^.

### Gap junctional coupling promotes the transdifferentiation of MSCs

GJs are intercellular structures that allow direct transfer of signaling molecules to modulate cellular processes in the recipient cell^19, 44^. FRAP microscopy indicated that MSCs interacted with neighboring cardiac cells via gap junctional cell-cell contacts, accompanied by the expression of Cx43 *in vitro* and *in vivo* (Fig. 5). Our data are in line with several studies that demonstrated the presence of cell-cell contacts and gap junctional coupling between BM derived MSCs and CMs^17, 45, 46^. This emphasizes the capability of MSCs to be successfully integrated into the tissue following transplantation, which is further supported by our qRT-PCR data, showing the presence of MSCs three weeks post transplantation.

Importantly, we found that the transdifferentiation process of MSCs was more efficient when gap junctional cell-cell contacts with CMs were established (Fig. 6), which corresponds to previous reports defining a role of GJs in the cardiogenic differentiation of MSCs^15^. However, published studies provide only little information regarding the potential mechanism through which MSC transdifferentiation is induced by GJIC with myocytes. We hypothesize that CM-derived miRNAs are transferred via GJs to facilitate the cardiac differentiation of co-cultured MSCs^47^. Indeed, our results revealed that small non-coding RNAs can be shuttled from CMs to MSCs in a Cx43 dependent manner leading to gene regulatory effects (Fig. 7). Additionally, impairment of the miRNA machinery in CMs decreased the respective expression of NKX2.5 in MSCs (Fig. 7). The ability of MSCs to participate in the gap junctional transfer of miRNA was previously reported for other co-culture systems, e.g. cancer cells^48–50^. Moreover, cardiogenic miRNAs, like miR-499 and miR-133 have been demonstrated to stimulate the cardiac differentiation of MSCs, including up-regulation of NKX2.5 and GATA-4^51, 52^. Future studies are needed to identify the miRNA candidates involved in the lineage specification of MSCs, which can be further used for their preconditioning and thereby promoting the cardiogenic potential^53^.

The integration of MSCs into the cardiac environment by gap junctional cell-cell contacts also allows the translocation of Ca^2+^ ions^54^. In this study, we observed changes of the Ca^2+^ level in co-cultured MSCs. However, it remains to be determined whether these Ca^2+^ oscillations are based on the translocation of Ca^2+^ from adjacent CMs or on intrinsic events induced by CMs (Fig. 5). The first hypothesis is supported by a study of Muller-Borer and colleagues on co-culture of CMs and liver stem cells. They demonstrated a diffusion of Ca^2+^ ions from myocytes into stem cells which resulted in Ca^2+^ oscillations^55^. Interestingly, these oscillations were closely related to the induction of NKX2.5, TBX5 and myocardin in the co-cultured stem cells, while its inhibition abolished the cardiac specific gene program.

### GJs as a target for cell engineering

Multiple independent preclinical studies employed the concept of *ex vivo* stimulation of BM derived cells with various factors in order to achieve cardiac specification of these cells prior to transplantation^7, 10, 56, 57^. A subsequent clinical trial relying on this approach has proven safety and efficacy of such “next generation” MSCs, which prior to their transplantation had been exogenously stimulated with cytokines to induce differentiation into cardiac progenitors^58^.

Our study shows that MSCs possess an intrinsic ability to acquire a cardiac-like phenotype, yet this process is greatly enhanced by gap junctional coupling with surrounding myocytes (Fig. 5). Thus, we suggest GJs as a promising target for cell engineering of MSCs to increase their regenerative potential. Previous studies indicated that the host myocardium can provide factors capable to facilitate the differentiation process of stem cells after transplantation^16, 59, 60^. MSCs could be modified in a way that increases their coupling activity e.g., by up-regulation of Cx43, which in turn would enhance their ability to receive cardiogenic cues from the host tissue. Furthermore, GJIC reversely also offers the possibility to influence the cardiac microenvironment by stem cell application. In this regard, it was postulated that GJs can provide a pathway to deliver therapeutic molecules to adjacent cells^47, 61^. As Hahn and co-workers demonstrated, an increase in GJIC via stimulation by different growth factors mediated the anti-apoptotic effects of transplanted MSCs on the infarcted myocardium^62^.

Possibly even more importantly, MSCs can be loaded with therapeutic molecules and used as vectors for their delivery into the damaged heart tissue^61, 63, 64^. In this concept, facilitated molecular exchange with the surrounding tissue after transplantation would be beneficial.

To summarize, our findings demonstrate a beneficial improvement of co-transplantation (vs single treatment) of 2 distinct stem cell types with different regenerative mechanisms. Further, *in vitro* and *in vivo* investigation of direct interaction between transplanted cells and host myocardium confirmed the establishment of functioning GJIC by MSCs as opposed to HSCs. This intercellular crosstalk promotes activation of the intrinsic program of cardiac lineage specification by the transfer of cardiogenic signals from adjacent CMs, e.g. small RNAs. However, to further support our hypothesis, future *in vivo* investigations need to be performed. This would include the transplantation of modified MSCs with reduced or increases gap junction activity, followed by cell fate analysis. In addition, a gap junction blocking approach *in vivo* would facilitate extrapolation of our *in vitro* data to *in vivo* conditions.

Nevertheless, our data suggest that GJs are promising targets for MSC preconditioning to further promote their cardiogenic differentiation and to improve their therapeutic activity. Moreover, since transplanted stem cells may also act as donor cells in gap junctional shuttling, manipulation of GJIC could be a powerful tool in cell engineering.

## Materials and Methods

### Bone marrow aspiration

BM was aspirated from informed donors who gave written consent to the use of their BM for research according to the Declaration of Helsinki. The ethical committee of the Medical Faculty of the University of Rostock has approved the presented study as of 2010 (registration no A 2010 23; renewal in 2015). BM samples were obtained by sternal aspiration from patients undergoing coronary artery bypass graft surgery at Rostock University Medical Center. Anticoagulation was achieved by heparinization with 250 i.E./ml sodium heparin (Ratiopharm).

### Human cell isolation

Mononuclear cells were isolated by density gradient centrifugation on 1077 Lymphocyte Separation Medium (LSM; PAA Laboratories). CD133^+^ HSCs and CD271^+^ MSCs were enriched by positive magnetic selection using the MACS cell separation system. In case of CD133^+^ direct labeling was applied and indirect labeling for CD271^+^ cells (CD271-APC/anti-APC-microbeads) (all from Miltenyi Biotec). Cells were processed in phosphate buffered saline (PBS) containing 0.5% bovine serum albumin (BSA) and 2 mM EDTA (both from Sigma-Aldrich). Purity and viability of all cell isolations were verified by flow cytometric method.

### Cell culture and CM isolation

Human stem cells were cultured at 5% CO_2_ and 37 °C in MSC growth medium supplemented with SingleQuot (all Lonza) and 100 u/l penicillin and 100 µg/ml streptomycin (PAN Biotech). Medium was changed twice a week. All experiments involving neonatal mice were performed according to the ethical guidelines for animal care of the Rostock University Medical Centre. CMs from neonatal NMRI mice were isolated using Pierce primary CM isolation kit according to the manufacturer’s instructions (Thermo Fisher Scientific). Briefly, isolated hearts were digested with an enzyme mix containing papain and thermolysin for 35 min at 37 °C. Subsequently, cell suspension was plated on non-coated culture vessel to allow adherence of non-cardiomyocytes. After 2 hours the supernatant was centrifuged and cells were used for further experiments. CMs were cultivated in DMEM supplemented with 10% FBS and 1% P/S (all Pan Biotech) on 0.1%-gelatin (Sigma-Aldrich) coated surfaces. After one day a proliferation inhibitor (included in the CM isolation kit) was added to prevent extensive fibroblast growth. For co-cultures, CM and human stem cells were seeded at 1:80 ratio and cultured in DMEM, 10% FBS and 1% P/S.

### Fluorescence-activated cell sorting (FACS) analysis

The following mouse anti-human antibodies were applied in all experiments (with respective isotypes used as control): anti- CD133-phycoerythrin (PE) (293C2), anti-CD34-fluorescein isothiocyanate (FITC) and anti-CD271-allophycocyanine (all Miltenyi Biotec); anti-CD45-allophycocyanin- H7 (APC-H7), anti-CD45-Horizon V500, anti-CD29- allophycocyanin (APC), anti-CD44-Peridinin chlorophyll protein-Cyanine 5.5 tandem dye (PerCP-Cy5.5), anti-CD73-PE, anti-CD146-V450 (P1H12), anti-CD34-PE-Cyanine 7 (Cy7) as well as 7-aminoactinomycin (7-AAD) (all Becton Dickinson), anti-PDGFR-β-PE (18A2) (BioLegend) and anti-CD105-Alexa Fluor 488 (AF488) (AbD Serotec). Near IR live dead stain and 4’,6-Diamidino-2-phenylindole (DAPI) were obtained from Thermo Fisher Scientific.

Cells were suspended in PBS containing 0.5% BSA and 2 mM EDTA and incubated with FcR blocking reagent (Miltenyi Biotec) for 10 min in the dark at 4 °C to reduce unspecific binding. Samples were treated with erythrocyte lysis solution containing 150 mM ammonium chloride, 10 mM potassium hydrogen carbonate and 100 µM EDTA (all Sigma-Aldrich), pH 7.27, followed by incubation for 10 min on ice. Finally, samples were analyzed with LSRII flow cytometer and FACSDiva software version 6.1.2 (Becton Dickinson). Compensation was established using single stained controls and gating was performed with matched isotype/fluorescence minus one (FMO) controls. The Boolean gating strategy for CD133^+^ cells was modeled based on the International Society of Hematotherapy and Graft Engineering (ISHAGE) guidelines for CD34^+^ cell analysis. CD271^+^ cells were gated based on viable SSC^low^ cells.

### Colony forming unit-fibroblast assay (CFU-F) and Hematopoietic colony forming unit assay (CFU)

Freshly isolated MSCs and HSCs (40,000 CD271^+^ or CD133^+^ cells per well; 1 × 10^6^ mononuclear cells or CD271^−^ cells per well) were seeded into 6 well plates (Greiner bio-one) and incubated in 2 mL complete MSCGM medium. Medium was changed once after 7 days. CFU-F were counted microscopically after 14 days, fixed by incubation with methanol (Sigma-Aldrich) for 5 min at room temperature and subjected to Giemsa staining to enhance visibility (Sigma-Aldrich) according to the manufacturer’s instructions.

For CFU freshly isolated cells (5000 CD133^+^ or CD271^+^ cells per dish) were subjected to a hematopoietic colony forming unit-assay (MethoCult H4434 Classic, Stemcell Technologies) following manufacturer’s protocol. Colonies were counted after 14 days and differentiated into colony forming unit-erythroid (CFU-E), burst forming unit-erythroid (BFU-E), colony forming unit-granulocyte monocyte (CFU-GM) and colony forming unit-granulocyte, erythrocyte, monocyte, megakaryocyte (CFU-GEMM).

### Experimental design of the animal model

All experiments on mice were carried out in accordance with German legislation and the EU-directive 2010/63/EU and were approved by the federal animal care committee Landesamt für Landwirtschaft, Lebensmittelsicherheit und Fischerei Mecklenburg-Vorpommern (approval number LALLF M-V/TSD/7221.3-1.1-088/11). Severe Combined Immunodeficiency beige mice (SCID *beige*; strain CB17.Cg-*Prkdc* ^*scid*^ *Lyst* ^*bg*−*J*^/Crl, female, 22 ± 2 g, Charles River) were randomly assigned to 5 groups: healthy control (Sham, n = 7), three MI groups with implanted human stem cells of the respective source (MI133, MI271, MIX, each n = 7) and untreated MI control group (MIC n = 8).

### Generation of reperfused MI in mice and stem cell implantation

Mice were anesthetized with 50 mg/kg Pentobarbital intraperitoneal injection. After thoracotomy and preparation, the left anterior descending coronary artery (LAD) was ligated. After 45 min each mouse received an intramyocardial cell injection. For cell treatment, 10^5^ stem cells were suspended in 10 µl PBS containing 0.5% BSA and 2 mM EDTA and mixed with an equal amount of reduced growth-factor BD Matrigel^TM^ Matrix (Becton Dickinson). The same experimental set-up was applied to control groups using cell suspension buffer and Matrigel^TM^ for application. Injections of 4×5 µl were given along the border of the blanched myocardium. Subsequently, the ligation was removed. SHAM-operated mice underwent identical surgical procedures without LAD-ligation.

### Analysis of cardiac functions

Pressure-volume (PV)-loop measurements were conducted 3 weeks after surgery according to the protocol of CardioDynamics BV (CD Leycom). Data were collected with the Millar Pressure-Volume System (Ultra-Miniature Pressure-Volume Catheter (model SPR-1030), Millar Pressure Conductance Unit (model MPCU-200) and Millar PowerLab data-acquisition hardware (Emka Technologies). Calibration of pressure and volume was performed by equating the minimal and maximal conductance with minimal (0 mmHg) and maximal (100 mmHg) pressures as well as minimal and maximal blood volumes received from venous circulation. After inserting the catheter into the carotid artery, retrograde access to the left ventricle was achieved. Volume signal was corrected by measurement of wall conductance (parallel volume) via hypertonic saline (5%) injection. Data were analyzed with IOX Version 1.8.3.20 software (Emka Technologies). After PV-loop measurements blood vessels were stained with 7.5 µg/ml of biotinylated *Lycopersicon esculentum* (Tomato) lectin (LINARIS) by perfusion of the venous circulation for 10 min. Euthanasia was performed by heart arrest in diastole with potassium chloride.

### Organ harvesting

Each heart was removed, embedded in O.C.T.^TM^ Compound (Tissue-Tek^®^) and snap-frozen in liquid nitrogen. For histological and biomolecular investigations, the whole infarct area of the heart tissue was divided into 4 horizontal levels and cut into sections of 5 µm thickness. The three interlayers between the levels were collected at −80 °C for RNA isolation. Lungs were snap-frozen in liquid nitrogen and stored at −80 °C for RNA isolation.

### Fibrosis analysis

Heart sections of 4 horizontal infarction levels (5 µm) were stained with Sirius Red (Division Chroma) to visualize collagen deposition and Fast Green FCF (Sigma-Aldrich) to display uninjured muscle tissue. Two contiguous levels of the heart which represent the major infarction ratio were analyzed using computerized planimetry (Axio Vision LE Rel. 4.5 software; Carl Zeiss). To evaluate fibrosis, the Sirius Red positive regions of collagen deposition in the infarction border zone were examined in 5 randomly chosen fields per section (one section per level; 400×) using computerized planimetry. Collagen density was expressed as the ratio of collagen deposition to myocardial tissue in percentage.

### Determination of blood vessels

To investigate the capillary density and angiogenesis, hearts were perfused with Tomato lectin. Heart sections of two contiguous levels of the heart which represent the major infarct region were fixed with 2% PFA and immunostained with polyclonal goat anti-Biotin (Vector Laboratories) primary antibody followed by incubation with anti-goat Alexa-Fluor^®^ 488 (Thermo Fisher Scientific) conjugated secondary antibody. Nuclei were counterstained with DAPI (Sigma-Aldrich). The sections were analyzed within the border zone and infarcted scar of the heart. Capillary density and neovascularization were assessed by counting the number of capillaries in 5 randomly chosen fields of the border zone and infarcted scar per section (one section per level; 400×). Results were expressed as capillaries per high power field (HPF).

### RNA isolation and polymerase chain reaction (PCR)

To determine the detection threshold for human cells in murine tissue 1 × 10^3^, 1 × 10^4^ and 1 × 10^5^, stem cells and murine heart tissue were lyzed in TRIzol ^®^ reagent (Thermo Fisher Scientific). RNA was isolated following the instructions of the TRIzol ^®^ standard protocol. In order to identify the amount of implanted human cells in murine tissue total RNA was isolated from the collected lungs and from interlayers of cryosectioned hearts using TRIzol ^®^ reagent. For reverse transcription of total RNA amount (2 µg) and cDNA synthesis, SuperScript^®^ III Reverse Transcriptase (Thermo Fisher Scientific) and oligo (dT)_15_ Primers (Promega) were used.

For the detection of NKX2.5 and GATA-4 levels in MSC-CM co-cultures RNA was isolated using DirectZol^TM^ RNA MiniPrep isolation kit (Zymo Research) according to the manufacturer’s instructions. For reverse transcription of 10 µl isolated RNA High-Capacity cDNA Reverse Transcription Kit (Thermo Fisher Scientific) was applied.

Quantitative real time-PCR was performed with StepOnePlus^TM^ Real-Time PCR System (Applied Biosystems) in TaqMan^®^ Universal Master Mix (Thermo Fisher Scientific) according to the instructions of the manufacturer. The following program was used: 1 cycle of 50 °C for 2 min, 1 cycle of 95 °C for 10 min, and 40 cycles of 95 °C for 15 s and 60 °C for 1 min. Human GAPDH (TaqMan^®^ Gene Expression Assay ID: Hs99999905_m1) was tested in duplicate and normalized against murine GAPDH (Endogenous Control: 4352339E). Similarly, human NKX2.5 (TaqMan® Assay ID: Hs03988822_m1) and human GATA-4 (TaqMan® Assay ID: Hs00171403_m1) were analyzed in duplicates and normalized to human HPRT (TaqMan® Assay ID: Mm00446968_m1). Negative controls were included in each assay. Cycle thresholds (C_T_) for single reactions were determined with StepOne™ Software 2.0 (formula: ΔC_T mean_ = C_T mean hGAPDH_ − C_T mean mGAPDH_).

### Transfection

siRNA-mediated knockdown of Cx43 and Dicer was performed by electroporation with Amaxa nucelofector II (Lonza) according to the manufacturer’s instructions. Cells were harvested and resuspended in nucleofection buffer containing 90 mM Na2PO4, 90 mM NaH_2_PO_4_, 5 mM KCl, 10 mM MgCl_2_ and 10 mM sodium succinat. 250 nM Cx43, Dicer siRNA or control siRNA (Sigma Aldrich) were transfected using nucleofector program U-23 for MSCs or G-009 for CMs. If specified, MSCs were transfected with 3 µg EGFP Plasmid. To study miRNA exchange between CMs and MSCs, CMs were transfected with 250 nM DY-547 labeled control miRNA (Dharmacon).

### Immunofluorescence staining

For immunofluorescence staining of co-cultures, cells were grown on glass coverslips and fixed with 4% paraformaldehyde (Sigma Aldrich) for 20 min at room temperature. Following a permeabilization step with 0.1% Triton X-100 for 5 min (Sigma Aldrich), cells were incubated with 1% BSA to reduce unspecific antibody binding. For expression analysis of cardiac transcription factors, cells were labeled with goat anti NKX2.5 (Santa Cruz), rat anti GATA-4 (ebioscience) and mouse anti-human nuclei (Merck Millipore). Subsequently, cells were incubated with donkey anti-goat Alexa Fluor488^®^, donkey anti-rat Alexa Fluor647® and donkey anti-mouse Alexa Fluor594^®^ conjugated secondary antibodies (all Thermo Fisher Scientific). To visualize α-actinin and GJs, co-cultures were labeled with mouse anti α-actinin and rabbit anti Cx43, followed by incubation with goat anti-mouse Alexa Fluor488^®^ and goat anti-rabbit Alexa Fluor594^®^ conjugated secondary antibodies. Nuclei were stained with DAPI and cells were mounted on microscope slides using Dako mounting medium (Dako).

Crysectioned hearts were treated with M.O.M.^TM^ Mouse Ig Blocking Reagent and M.O.M.^TM^ protein concentrate following the instructions of Vector^®^ M.O.M.^TM^ Immunodetection Kit (LINARIS). To identify human cells, monoclonal anti-human-nuclei (Merck Millipore) primary antibody and anti-mouse Alexa-Fluor^®^ 594 was used. Cx43, NKX2.5 and GATA-4 were visualized with primiary antibodies as described above.

### Confocal microscopy and image analysis

Fluorescence images of fixed cells were obtained using a Zeiss ELYRA PS.1 LSM 780 confocal imaging system with 60x/NA 1.3 or 40x/NA 1.3 oil objectives. Images were acquired from z-stacks of 20 images and maximum projections were created using Zen2011 (Carl Zeiss). For quantification of NKX2.5 and GATA-4 levels, parameters (e.g., gain, laser power, offset) were kept constantly throughout one experiment (control vs. treatment).

To quantify the expression of NKX2.5 and GATA-4 in MSCs, maximum projections of z-stacks were created and images were analyzed by Zen2011 software. Nuclei were selected manually and the integrated mean fluorescence was calculated (mean fluorescence × nuclei size) and compared with corresponding control to obtain fold change values.

### Fluorescence Recovery after Photobleaching (FRAP) microscopy

The extent of gap junctional coupling between MSCs, HSCs, and CMs was measured by FRAP microscopy. For identification, stem cells were labeled with 5 µM Vybrant® CFDA SE cell tracker (Thermo Fisher Scientific) for 15 min before co-cultured with CMs on 4-chamber µ-slides (ibidi). After 4 days cells were loaded with 5 µM calcein red orange (Thermo Fisher Scientific) for 30 min at 37 °C and washed with PBS. 3D-FRAP measurement was performed using ELYRA PS.1 LSM 780 confocal imaging system equipped with a CO_2_ cage incubator (Carl Zeiss). Images were acquired with 40x/NA 1.3 oil objective. A single MSC within a cluster of CMs was bleached for 50 s by a 561 nm laser beam. Fluorescence recovery was detected every 60 s for 13 min by z-stack acquisition from 15 images. For image analysis maximum projections were created using Zen2011 software (Carl Zeiss). Two unbleached cells were selected as reference to exclude photobleaching artefacts during FRAP measurement. All FRAP measurements were performed on 6–18 cells per experiment and repeated at least 4 times.

### Calcium Imaging

For calcium imaging, cells were loaded with 4 µM X-Rhod-1 (Thermo Fisher Scientific) for 60 min at 37 °C, washed 3 times with medium and analyzed using a Zeiss ELYRA PS.1 LSM 780 confocal imaging system. Measurements were performed at 37 °C and 5% CO_2_. Quantification of the changes of fluorescence intensity of X-Rhod-1 were achieved by analyses of 5 circular ROIs in each cell for 30 s. Amplitudes of intensity changes were calculated at 1; 5; 10; 15; 20; 25 and 30 s of each time frame.

### Live cell measurement of fluorescence intensity

EGFP expression in MSCs was measured with Infinite 200 PRO microplate reader (Tecan). Following transfection with EGFP, anti-EGFP siRNA or control siRNA, cells were seeded on flat bottom 96 well plates (Greiner bio-one) and cultured for another 2 days. For the measurement of EGFP intensity medium was exchanged with PBS. Measurements were performed as triplicates and with 9×9 multiple reads per well.

### Statistical analysis

All values are presented as mean ± standard error of the mean (SEM). Statistical significance between two groups was calculated using two-tailed Student’s t-test. FRAP data were analyzed by two-way analysis of variance (ANOVA), including Bonferroni’s post hoc test. For multiple comparisons one-way ANOVA was applied, followed by Tukey’s post hoc test. Gaussian distribution was analyzed using Pearson normality test and Kolmogorov-Smirnov test. Kruskal-Wallis test was performed when no normal distribution was assumed, followed by Dunn’s post hoc test. Probability levels considered as statistically significant were *P ≤ 0.05, **P ≤ 0.01 and ***P ≤ 0.001. Calculations and graph analysis were composed with GraphPadPrism5 software (GraphPad Prism, Inc.).

### Data availability

The data acquired for this study are available within supplementary files and from the corresponding author on reasonable request.

## Electronic supplementary material

Supplementary Video 1

Supplementary Video 2

supplemental data
