# Supplementary material for: Mechanisms of stem cell based cardiac repair-gap junctional signaling promotes the cardiac lineage specification of mesenchymal stem cells
Source: Sci Rep. 2017 Aug 29;7:9755. doi: 10.1038/s41598-017-10122-6 (PMC5574972; doi:10.1038/s41598-017-10122-6)
Supplement: Supplementary file 3 — supplemental data [file 41598_2017_10122_MOESM3_ESM.pdf]

Supplemental Data

Mechanisms of stem cell based cardiac repair - gap junctional signaling promotes the cardiac lineage specification of mesenchymal stem cells

Heiko Lemcke[1,2*#], Ralf Gaebel[1,2*], Anna Skorska[1,2], Natalia Voronina[1], Cornelia Aquilina Lux[1], Janine Petters[1], Sarah Sasse[1], Nicole Zarniko[1], Gustav Steinhoff[1,2], Robert David[1,2]

[1] Reference- and Translation Center for Cardiac Stem Cell Therapy (RTC), Department of Cardiac Surgery, University of Rostock, Schillingallee 69, 18057 Rostock, Germany

[2]Faculty of Interdisciplinary Research, Department Life, Light & Matter, University Rostock, Albert-Einstein Str. 25, 18059 Rostock, Germany

a

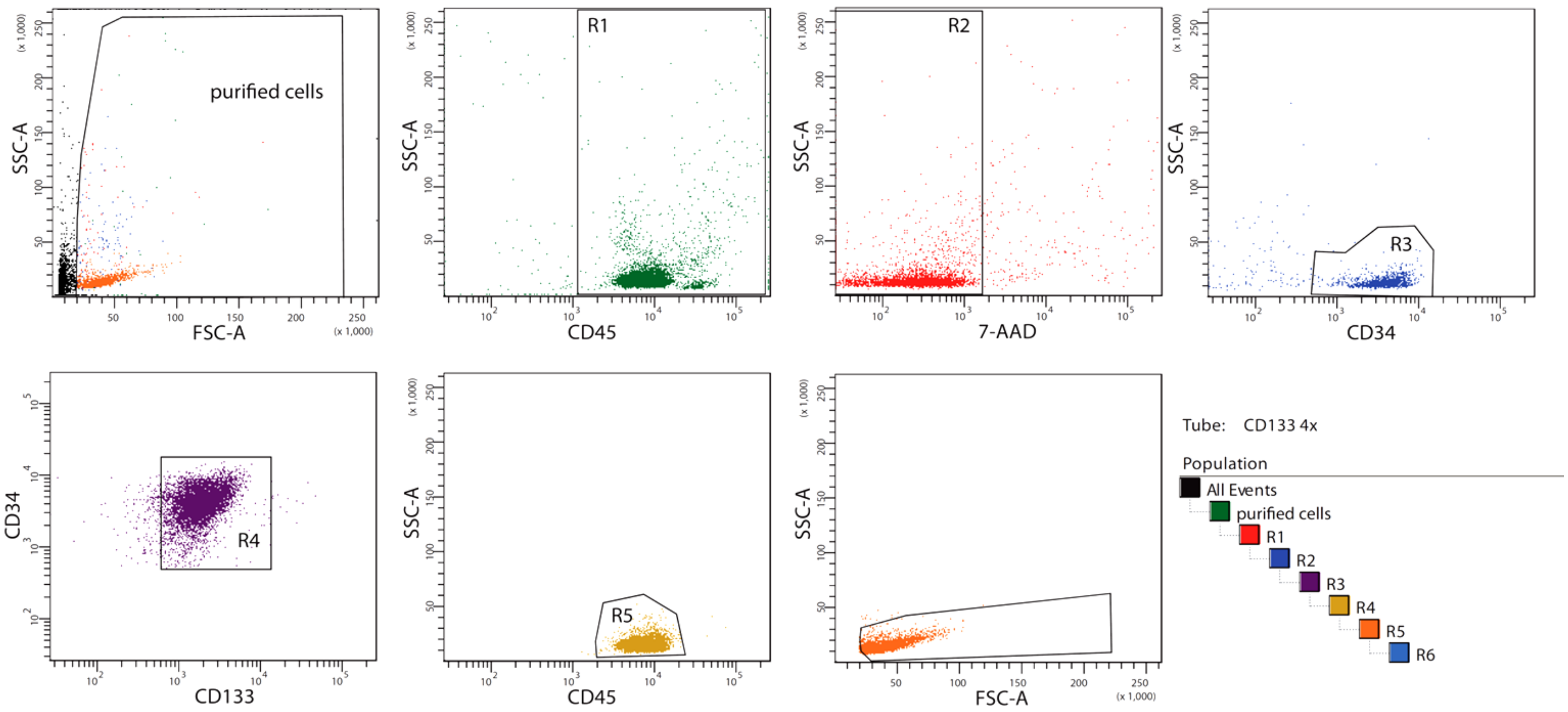
purified cells
SSC-A
FSC-A
R1
CD45
R2
7-AAD
R3
CD34
R4
CD133
R5
Tube: CD133 4x
Population
All Events
purified cells
R1
R2
R3
R4
R5
R6

b

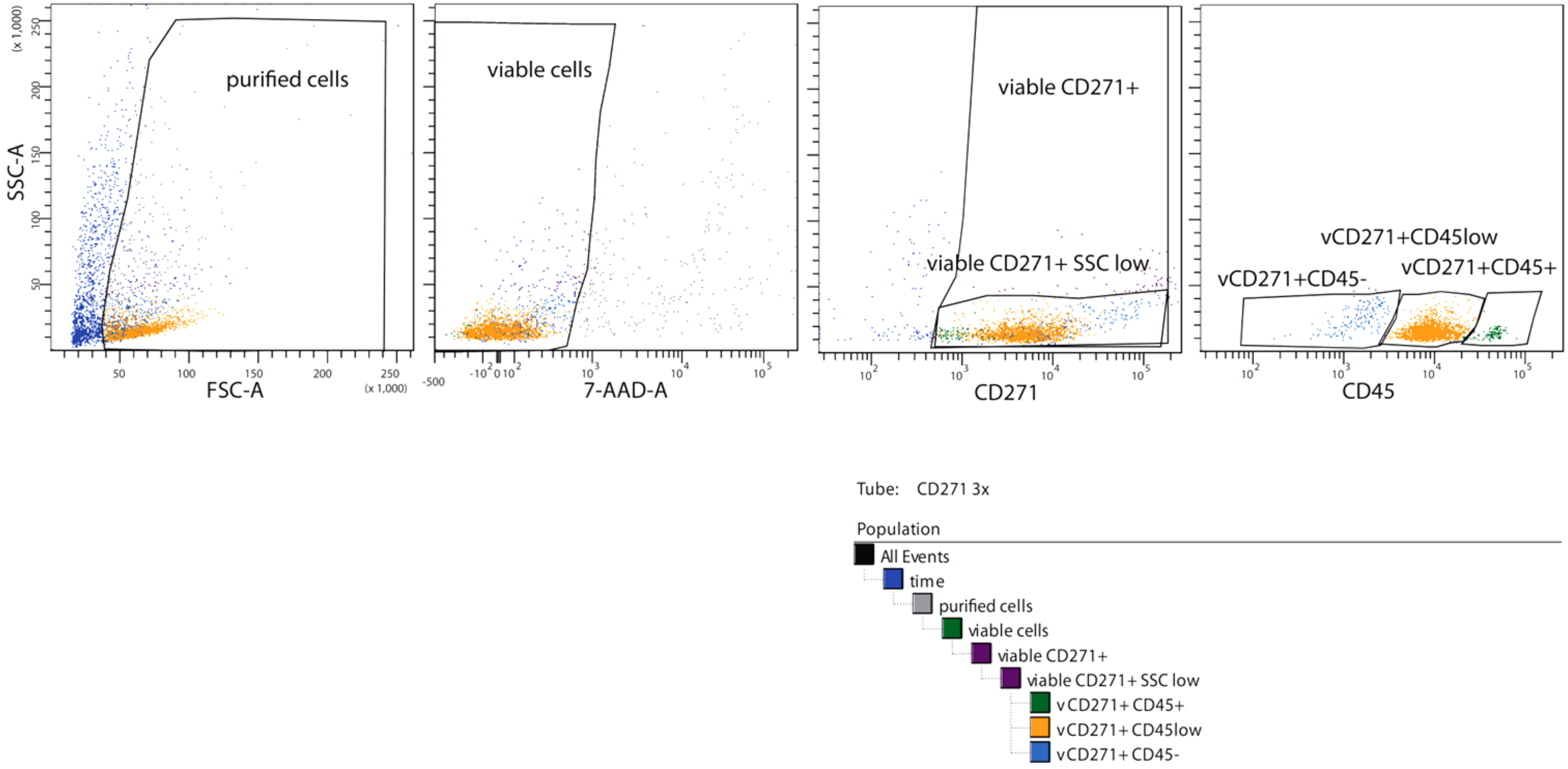

purified cells
SSC-A
FSC-A
(x 1,000)
viable cells
7-AAD-A
viable CD271+
viable CD271+ SSC low
CD271
vCD271+CD45low
vCD271+CD45-
vCD271+CD45+
CD45
Tube: CD271 3x
Population
All Events
time
purified cells
viable cells
viable CD271+
viable CD271+ SSC low
vCD271+ CD45+
vCD271+ CD45low
vCD271+ CD45-

Supplementary figure S1: Representative flow cytometric analysis of immunomagentic-based isolated CD133$^+$ HSCs and CD271$^+$ MSCs, respectively. ISHAGE based gating strategy was used for the qualitative analysis of isolated CD133$^+$ stem cells (a). Debris was excluded from CD45$^+$ cells (purified cells --> R1 (=all CD45$^+$) --> R2 (=viable CD45$^+$ cells)). CD34$^+$ cells were selected from viable CD45$^+$ cells (=R3). With the region R4 viable CD45$^+$CD34$^+$CD133$^+$ cells were selected and events with high expression of the CD45 marker were further excluded (R5). FSC/SSC backgate was employed to select viable CD45$^+$CD34$^+$CD133$^+$ cells with blast morphology (R6). Purity of manually isolated CD133$^+$ stem cells was calculated as follows: [=R6 events/R2 events × 100%]. For qualitative flow cytometric analysis of CD271$^+$ MSCs (b) debris/dead cells were excluded (region ‘purified cells’ --> region ‘viable cells’). Purity of MACS-isolated CD271$^+$ was calculated: [= ‘viable CD271$^+$ cells’/ ‘viable cells’ × 100 %]. Furthermore, viable CD271$^+$ MSCs with lower granularity (side scatter, SSC) were characterized for CD45 expression: lack CD45 (vCD271$^+$CD45$^-$), with lower expression of CD45 (vCD271$^+$CD45^{low}) and those with high expression of CD45 marker (vCD271$^+$CD45$^+$); v=viable.

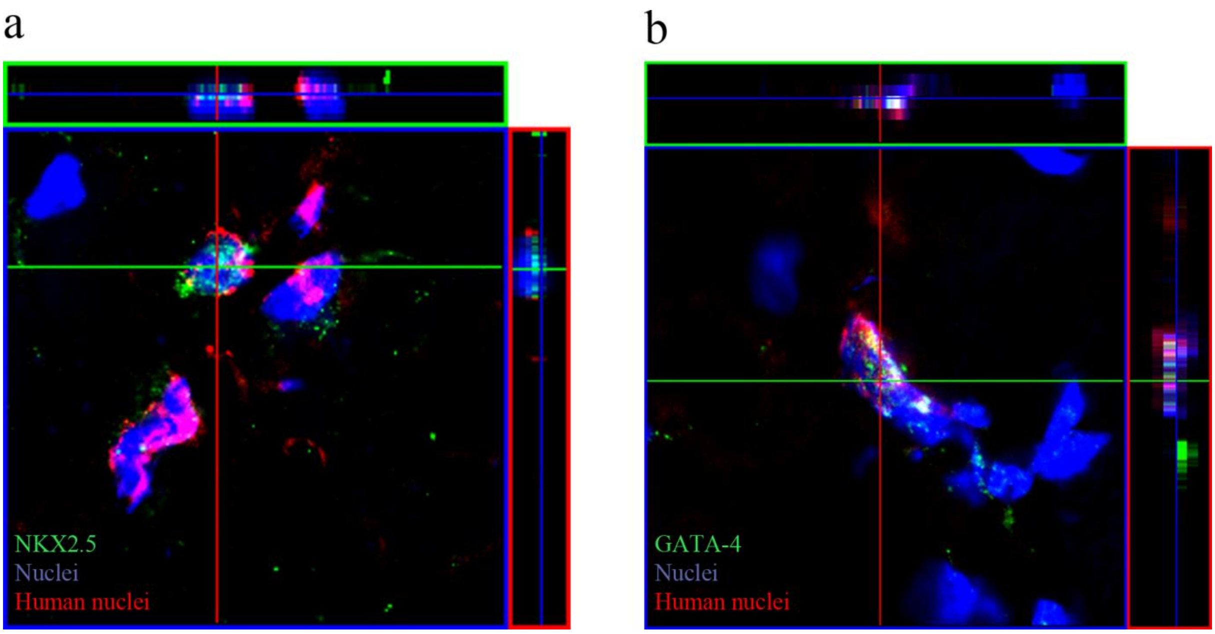

Supplementary figure S2. *In vivo* expression of early cardiac markers NKX2.5 and GATA-4 in mesenchymal stem cells, injected into murine heart. Orthographic projection of immunolabelled cryosections demonstrates the localization of (a) NKX2.5 and (b) GATA-4 protein within the nuclei of mesenchymal stem cells three weeks after transplantation.

1	2	3	4	5	6	7	8
$1x10^3$ cells 0hrs	$1x10^4$ cells 0hrs	$1x10^5$ cells 0hrs	Neg. control (no cells)	MSC 48hrs	HSC 48hrs	MSC 3 weeks	HSC 3 weeks
16.39	13.85	9.19	n.d	14.46	12.43	16.99	13.64
15.94	9.04	9.06	n.d	14.04	12.73	15.21	12.03
15.75	12.43	9.18	n.d.	9.18	15.81	16.91	13.09
21.10	16.49	10.22	n.d.	11.63	11.01	15.91	13.06
20.66	16.05	9.45	n.d	12.02	10.64	14.52	8.32
20.55	14.27	9.83	n.d	10.58	10.02	17.90	

Supplementary table S1: delta C_t values of performed qRT-PCRs to detect human GAPDH mRNA. Columns 1-3 demonstrate qRT-PCR results of one murine heart to which a certain number of stem cells was added (see Figure 4a). No amplification signal was detected in murine hearts that lack stem cell injection (negative control). Columns 5-8 show qRT-PCR data from murine hearts undergone stem cell treatment (see Figure 4b).

see supplementary movie S1

Supplementary movie S1. Calcium imaging in mesenchymal stem cell-cardiomyocyte co-culture. Left image shows GFP labelled stem cell, middle image corresponds to X-Rhod-1 channel, left image represents the overlay. Following loading with X-Rhod-1 cardiomyocytes showed oscillating fluorescence signal, caused by changes of the intracellular calcium level.

Slight, but significant alterations of the fluorescence intensity were also observed in GFP-transfected mesenchymal stem cells.

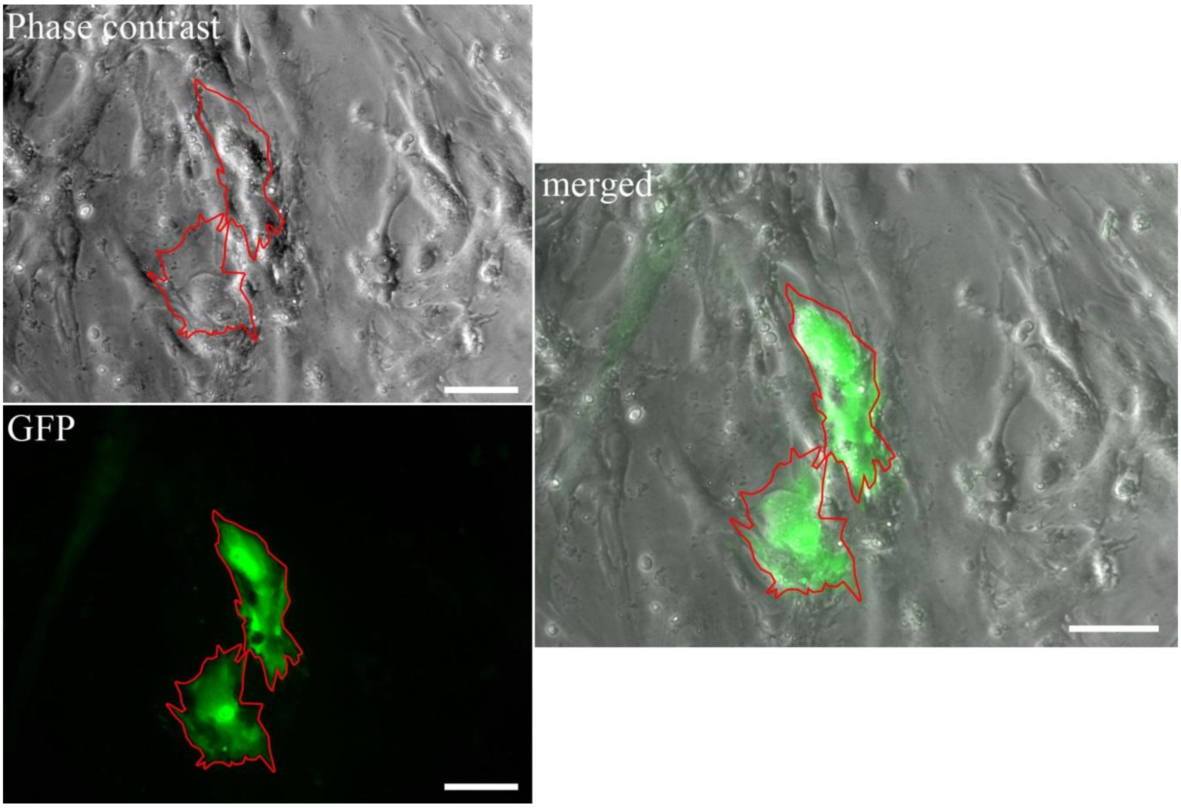

Supplementary figure S3. Co-culture of GFP-transfected mesenchymal stem cell and mice cardiomyocytes. Images show integration of stem cells into a cardiomyocytes mono layer. Corresponding time-lapse imaging of GFP-tagged mesenchymal stem cells is shown in movie S2. Scale bar 50 µm.

see supplementary movie S2

Supplementary movie S2. Co-culture of GFP-transfected mesenchymal stem cell and mice cardiomyocytes. Stem cells demonstrate contraction, consistent with the beating activity of adjacent cardiomyocytes.

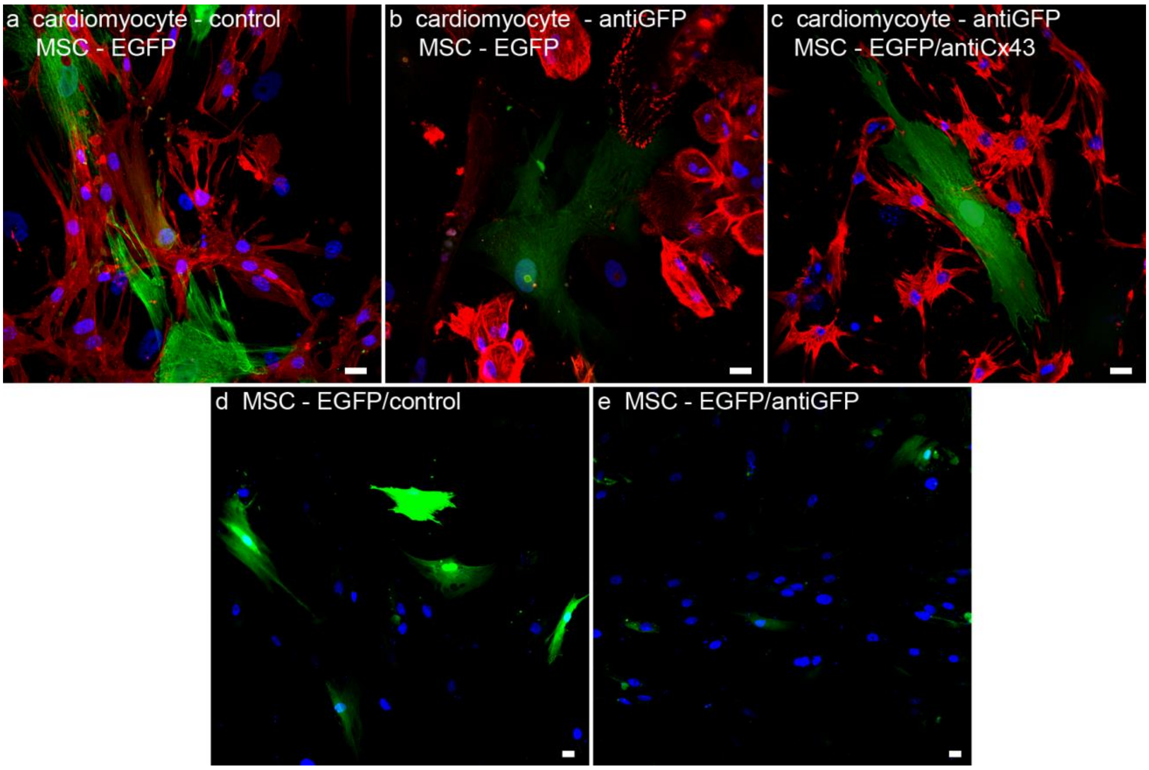

Supplementary figure S4. Cardiomyocyte derived small non-coding RNAs induce effects in recipient mesenchymal stem cells. Representative confocal images of cardiomyocyte and mesenchymal stem cell (MSC) co-cultures. Cardiomyocytes were transfected either with control siRNA (a) or anti-EGFP siRNA (b, c) and co-cultured with MSCs transfected with EGFP plasmid alone (a, b) or in combination with anti-Cx43 siRNA (c). The efficiency of the EGFP/siRNA reporter construct was evaluated by double transfection of MSC monoculture with EGFP and anti EGFP siRNA (e), leading to reduced GFP fluorescence. Scale bar 20 µm. Cells were transfected and cultured as described in material and methods section. Cardiomyocytes were labelled with anti-α-actinin antibody, nuclei were stained with DAPI.
